# Isolation and Characterization of New *Leptospira* Genotypes from Patients in Mayotte (Indian Ocean)

**DOI:** 10.1371/journal.pntd.0000724

**Published:** 2010-06-22

**Authors:** Pascale Bourhy, Louis Collet, Sabine Clément, Michel Huerre, Patrick Ave, Claude Giry, François Pettinelli, Mathieu Picardeau

**Affiliations:** 1 Unité de Biologie des Spirochètes, Institut Pasteur, Paris, France; 2 Centre Hospitalier de Mayotte, Mayotte, France; 3 Unité de Recherche et d'Expertise Histotechnologie et Pathologie, Institut Pasteur, Paris, France; Cambridge University, United Kingdom

## Abstract

**Background:**

Leptospirosis has been implicated as a severe and fatal form of disease in Mayotte, a French-administrated territory located in the Comoros archipelago (southwestern Indian Ocean). To date, *Leptospira* isolates have never been isolated in this endemic region.

**Methods and Findings:**

Leptospires were isolated from blood samples from 22 patients with febrile illness during a 17-month period after a PCR-based screening test was positive. Strains were typed using hyper-immune antisera raised against the major *Leptospira* serogroups: 20 of 22 clinical isolates were assigned to serogroup Mini; the other two strains belonged to serogroups Grippotyphosa and Pyrogenes, respectively. These isolates were further characterized using partial sequencing of 16S rRNA and *ligB* gene, Multi Locus VNTR Analysis (MLVA), and pulsed field gel electrophoresis (PFGE). Of the 22 isolates, 14 were *L. borgpetersenii* strains, 7 *L. kirschneri* strains, and 1, belonging to serogoup Pyrogenes, was *L. interrogans*. Results of the genotyping methods were consistent. MLVA defined five genotypes, whereas PFGE allowed the recognition of additional subgroups within the genotypes. PFGE fingerprint patterns of clinical strains did not match any of the patterns in the reference strains belonging to the same serogroup, suggesting that the strains were novel serovars.

**Conclusions:**

Preliminary PCR screening of blood specimen allowed a high isolation frequency of leptospires among patients with febrile illness. Typing of leptospiral isolates showed that causative agents of leptospirosis in Mayotte have unique molecular features.

## Author Summary

Leptospirosis has been recognized as an increasing public health problem affecting poor people from developing countries and tropical regions. However, the epidemiology of leptospirosis remains poorly understood in remote parts of the world. In this study of patients from the island of Mayotte, we isolated 22 strains from the blood of patients during the acute phase of illness. The pathogenic *Leptospira* strains were characterized by serology and various molecular typing methods. Based on serological data, serogroup Mini appears to be the dominant cause of leptospirosis in Mayotte. Further molecular characterization of these isolates allowed the identification of 10 pathogenic *Leptospira* genotypes that could correspond to previously unknown serovars. Further progress in our understanding of the epidemiology of *Leptospira* circulating genotypes in highly endemic regions should contribute to the development of novel strategies for the diagnosis and prevention of this neglected emerging disease.

## Introduction

Leptospirosis, a zoonotic disease with a worldwide distribution, is an important emerging infectious disease [1]. Rodents are a main reservoir of the pathogenic agents of this disease, spirochetes of the genus *Leptospira*, excreting the bacteria in their urine. Humans are usually infected through contaminated water. This increasingly common disease affects impoverished populations from developing countries and tropical regions [2]. Leptospirosis is an endemic disease in rural regions of developing countries because of the exposure to a large number of animal reservoirs [3], [4]. Furthermore leptospirosis is an emerging health problem in urban slums where inadequate sanitation has produced the conditions for rat-borne transmission of the disease [5], [6], [7]. Outbreaks of leptospirosis are associated with heavy seasonal rainfall [5], [8], [9], [10] and extreme climatic events, such as hurricanes [6], [11] and El Niño [12], [13]. Over the last decade, outbreaks of leptospirosis were also associated to adventure tourism [14], [15]. More than 500,000 cases of severe leptospirosis are currently reported each year, with case fatality rates exceeding 10% [16]. However, its prevalence is still underestimated due to low awareness among the medical community and an absence of specific symptoms and readily available tests. In addition, some patients may also experience transient or mild manifestations [2].

**Figure 1:**
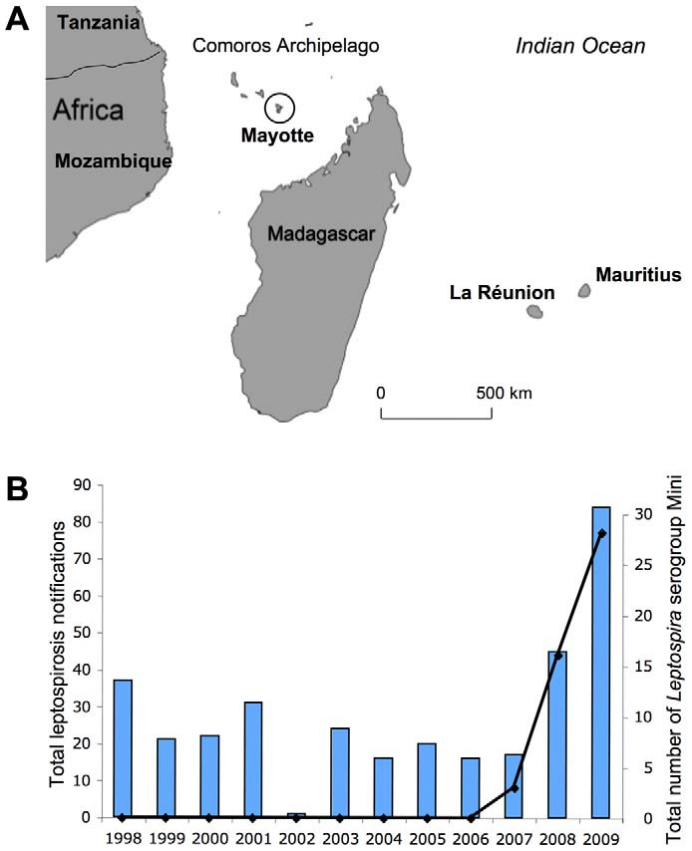
Leptospirosis in Mayotte. **A**. Location of Mayotte (circle) in the Comoros Archipelago. **B**. Distribution of cases of serogroup Mini compared with the total number of leptospirosis cases in Mayotte between 1998–2009.

Mayotte is a French-administrated territory located northwest of Madagascar in the Comoros archipelago, southwestern Indian Ocean (**Figure 1A**). The climate of this volcanic island is generally tropical and mild. The estimated population in 2007 was 186,387. The population is primarily of African origin. Unemployment is high and 39% of the population consists of immigrants from the neighboring Comoro Islands. Many of the inhabitants rear dairy herds and/or cultivate rice, cassava roots (manioc), maize, bananas or pineapples. Most households lack proper sanitation.

Leptospirosis is endemic in Mayotte [17]. The annual incidence of leptospirosis was previously estimated to be 3.8 patients per 100,000 individuals between 1984 and 1989 [17]. In 2008 and 2009, the annual incidence rate was estimated to be 45 patients per 100,000 individuals.

Leptospires are usually classified into species and serovars or serogroups. There are over 200 recognized pathogenic serovars; these serovars are currently clustered into 24 serogroups [1], [18], [19]. There have been no extensive studies on leptospiral serovars obtained from humans in Mayotte. Hence, we investigated the serological and genetic characteristics of leptospiral isolates isolated from leptospirosis patients in 2007–2008. Twenty-two plasma samples from heparinized blood specimens of individuals with leptospirosis-like illness testing positive for *Leptospira* spp. by PCR, were cultured and characterized by serology, sequencing of 16S RNA and *ligB*, and pulsed field gel electrophoresis (PFGE). In this study, our approach allowed a high rate of isolation of *Leptospira* from patients. We also report the existence of ten potentially new pathogenic *Leptospira* genotypes, which cause acute leptospirosis in Mayotte.

## Methods

### Isolation procedure of *Leptospira* spp

Blood samples (heparinized blood for culture and EDTA plasma for DNA extraction) were obtained from patients during the acute phase of illness (fever of 38°C or higher for no more than 7 days, accompanied by headache and/or myalgia) after oral assent after reading a script, which was approved by the Ethical Committee of the Centre Hospitalier de Mayotte, that informed of the possible use of blood samples for scientific purpose. Informed consent was recorded in writing in the patient's file as required by the Ethical Committee.

Ten drops (250 µl) and 20–40 drops (500–1000 µl) of plasma from heparinized blood were transferred into two tubes containing 9 ml of EMJH liquid medium [20], [21]. Cultures were incubated at 30°C and examined weekly, for 3 months, by dark field microscopy. In case of contamination, cultures were filtered through 0.22 µm pores to remove contaminants. Reference strains were obtained from the collection maintained by the National Reference Laboratory for *Leptospira*, which is also a WHO Collaborating Center, at the Institut Pasteur (Paris, France).

### Serogrouping

Serological characterization of clinical isolates was performed at the National Reference Center for *Leptospira*. A Microscopic agglutination test (MAT) was performed to determine the serogroup of *Leptospira* isolates using rabbit antisera against reference serovars representing a standard battery of 24 serogroups (Text S1). High rates of agglutination of the serum with one particular antigen are used to identify the presumptive serogroup of the infecting bacterium [22].

### Animal experiments

To determine if clinical isolates would induce an infection in laboratory animals, a group of four 28-day-old gerbils (Charles River Laboratories, http://www.criver.com) were inoculated intraperitoneally with 10^1^, 10^2^, 10^3^, 10^4^, and 10^6^ leptospires from strain 2007/01203. A group of control was also inoculated with EMJH medium. Animals were monitored daily for clinical signs of leptospirosis (i.e., prostration, jaundice, etc) and survival for up to 21 days post infection. Protocols for animal experiments were prepared according to the guidelines of the Animal Care and Use Committees of the Institut Pasteur.

Histopathologic analysis was perfomed after necropsy of infected animals which received 10^4^ leptospires at the day of death (6 or 7 days post -inoculation) and non infected animals. Liver, kidneys, and lungs were removed and fixed in 4% buffered formaldehyde for standard microscopic analysis; serial sections were stained with hematoxylin and eosin (HE) and Warthin–Starry silver impregnation as previously described [23]. The pathologist viewed the histopathological preparations without knowing the infection status of the animals.

### DNA manipulations

Genomic DNA was extracted from 400 µl of EDTA plasma using a MagNaPure Compact instrument (Roche Molecular Diagnostics), and yielded 50 µl of eluate. Leptospires in plasma were detected by quantitative real-time PCR (qPCR) using the Light cycler LC480 system (Roche) or the Cobas TacMan 48 system (Roche) as previously described [24]. A standard curve with DNA extracted from 10-fold dilutions of known numbers of leptospires was used for quantification. Samples with a threshold cycle (Ct) value >45 were considered negative. However, patient 13 with a Ct of 44.7 (**Table 1**) close to the cutoff was also included in this study.

**Table 1:** List of clinical isolates from Mayotte tested in this study.

patient	sex (M/F)	age (years)	Location	sampling date	Ct ^c^	bacteria/ml	strain	MAT ^d^	Species ^e^	*ligB*	VNTR					PFGE ^f^
											4	7	10	Lb4	Lb5	
Patient 1 ^a^	M	58	Dembeni	May 2007	28	1,0E+05	2007/01203 g	Mn 6,400	*L. borgpetersenii*	A	1	-	1	4	5	I
Patient 2 ^b^	M	23	Chiconi	Apr. 2007	34.9	6,7E+02	2007/01204	Mn 6,400	*L. borgpetersenii*	A	1	-	1	4	5	I
Patient 3 ^b^	M	21	Ouangani	May 2007	35.9	3,3E+02	2007/01473	Mn 3,200 Hb 800	*L. borgpetersenii*	A	1	-	1	4	5	I
Patient 4 ^b^	M	20	Bandraboua	Feb. 2008	34.9	6,7E+02	2008/01285	Mn 3,200	*L. borgpetersenii*	A	1	-	1	4	5	I
Patient 5	F	22	Mamoudzou	Feb. 2008	31.3	9,2E+03	2008/01286	Mn 3,200	*L. borgpetersenii*	A	1	-	1	4	5	I
Patient 6 ^a^	M	36	Dembeni	Feb 2008	25.2	7,7E+05	2008/01287	Mn 3,200	*L. borgpetersenii*	A	1	-	1	4	5	I
Patient 7 ^b^	M	23	Dembeni	Mar. 2008	28.1	9,3E+04	2008/01593	Mn 3,200	*L. borgpetersenii*	A	1	-	1	4	5	I
Patient 8	F	25	Chiconi	Mar. 2008	30.9	1,2E+04	2008/01594	Mn 6,400	*L. borgpetersenii*	A	1	-	1	4	5	I
Patient 9	M	32	Mamoudzou	Apr 2008	31.2	9,8E+03	2008/01924	Mn 12,800	*L. borgpetersenii*	A	1	-	1	4	5	I
Patient 10	M	39	Chiconi	Apr. 2008	30.9	1,2E+04	2008/01931	Mn 800	*L. borgpetersenii*	A	1	-	1	4	5	I
Patient 11	M	59	Chiconi	Jun. 2008	33.9	1,4E+03	2008/02842	Mn 12,800 Hb 800	*L. borgpetersenii*	A	1	-	1	4	5	I
Patient 12	M	20	Chirongui	Apr. 2008	38.1	6,6E+01	2008/01773	Mn 400	*L. borgpetersenii*	B	1	-	1	8	5	II
Patient 13	M	78	Mtsamboro	Apr. 2008	44.7	<1	2008/01929	Mn 400	*L. borgpetersenii*	B	1	-	1	8	5	II
Patient 14 ^b^	M	29	Tsingoni	Apr. 2008	37.9	7,6E+01	2008/01927	Mn 800	*L. borgpetersenii*	B	1	-	1	8	5	III
Patient 15	M	13	Chirongui	Apr. 2008	40.9	8,6E+00	2008/01926	Mn 800	*L. kirschneri*	C	0	2	1	ND	ND	IV
Patient 16	M	45	Sada	May 2008	37.5	1,0E+02	2008/02843	Mn 6,400 Hb 1,600	*L. kirschneri*	C	0	2	1	ND	ND	V
Patient 17	F	33	Tsingoni	Mar. 2008	35.7	3,8E+02	2008/01925	Mn 3,200	*L. kirschneri*	C	0	2	1	ND	ND	V
Patient 18	M	26	Dembeni	Dec 2007	38.3	5,7E+01	2008/00695	Mn 3,200 Hb 200	*L. kirschneri*	C	0	2	1	ND	ND	VI
Patient 19	F	29	Bandraboua	Apr. 2008	37.4	9,5E+01	2008/02841	Mn 6,400 Hb 6,400	*L. kirschneri*	C	0	2	1	ND	ND	VII
Patient 20 ^b^	M	52	Pamandzi h	Sep. 2008	37.6	1,1E+02	2008/03703	Mn 3,200 Hb 3,200	*L. kirschneri*	C	0	2	1	ND	ND	VIII
Patient 21	M	20	Tsingoni	Jan. 2008	40.6	1,1E+01	2008/01774	Gp 3,200	*L. kirschneri*	C	0	3	9	ND	ND	IX
Patient 22	F	39	Mtsamboro	June 2007	30.6	1,5E+04	2007/01872	Pyr 1,600	*L. interrogans*	D	6	10	6	ND	ND	X

ND: not determined.

a: lethal case associated with jaundice and acute renal failure.

b: patient with reported contact with water (swimming in a river) and/or animals (rats, dogs, cattle).

c: threshold cycle (Ct) value obtained by Real-Time PCR of DNA extracted from plasma samples.

d: Pyr, Pyrogenes; Mn, Mini; Hb, Hebdomadis (see Text S1 for the list of antisera used for serogrouping).

e: determined by 16S rRNA sequencing.

f: Pulsed field Gel Electrophoresis (PFGE) analysis with *Not*I.

g: strain used for animal infection experiments (see Figures 6 and 7).

h: probable acquisition of leptospirosis in Madagascar (see Discussion).

Genomic DNA was extracted from EMJH cultures using the Cell DNA Purification kit (Maxwell, Promega, Madison, WI). DNA was amplified using Taq polymerase (Amersham) under standard conditions. The amplified products were analyzed by 1% agarose gel electrophoresis. The 16S rRNA gene was amplified with the primers LA (5′-GGCGGCGCGTCTTAAACATG-3′) and LB (5′-TTCCCCCCATTGAGCAAGATT-3′) [25]. Partial *ligB* sequences were amplified with the primers PSBF 5′-ACWRVHVHRGYWDCCTGGTCYTCTTC-3′) and PSBR (5′-TARRHDGCYBTAATATYCGRWYYTCCTAA-3′) [26]. Sequencing was performed at the Genotyping of Pathogens and Public Health Platform (Institut Pasteur, Paris, France). The assembled sequence was then aligned against other 16S rRNA sequences available in GenBank using BLAST (http://www.ncbi.nlm.nih.gov/BLAST). Genotyping was also performed by multiple-locus variable-number tandem repeat analysis (MLVA) using the loci VNTR4, VNTR7, VNTR10, Lfb4 and Lfb5 as described by Salaun *et al.* [27].

For pulsed-field gel electrophoresis (PFGE), cells were embedded in agarose plugs as previously described [28]. DNA plugs were restriction digested with *Not*I. PFGE was performed in a contour-clamped homogeneous electric field DRIII apparatus (Bio-Rad Laboratories, Richmond, CA). Programs with a ramping from 1 to 70 s for 36 h at 150 V and from 10 to 100 s for 40 h at 150 V were used to resolve of restriction patterns.

## Results

### Sampling and culture of *Leptospira* isolates

**Figure 2:**
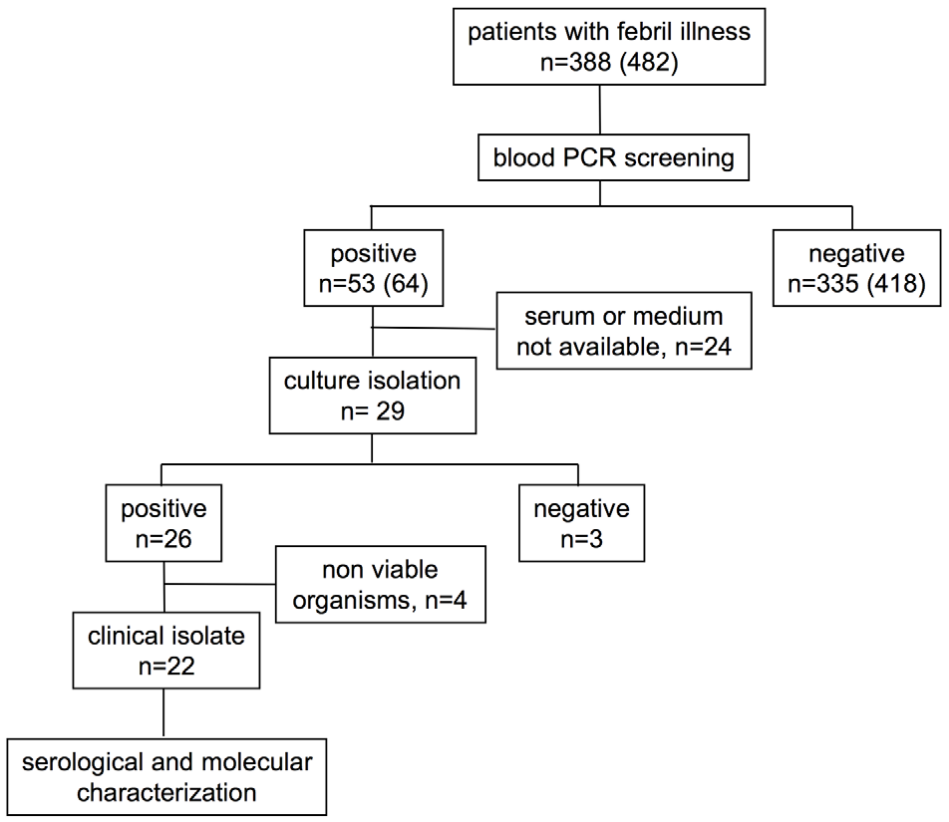
Flow diagram showing blood sample processing. In brackets the total number of serum samples, including serial samples.

We used blood sample cultures to investigate patients with febrile illness in Mayotte. We assayed 388 human plasma samples from blood collected in heparin by quantitative real-time PCR between April 2007 and September 2008 (not including serial samples from a same patient). The specific amplification of pathogenic *Leptospira* spp. was detected in 53 (13,7%) samples (**Figure 2**). Blood samples from 29 patients diagnosed with leptospirosis (as determined by PCR) were examined by culture. Samples from 26 patients (89.7%) were positive by culture, but leptospires in 4 cultures were lysed on arrival at the National Reference Center of Leptospira (Institut Pasteur). Twenty-two clinical isolates were therefore included in this study. The median time to culture positivity for the primary culture was 19.3 days (7 to 77 days).

Ct values of samples that were positive by culture were between 25.2 and 44.7 (median 34.9) (**Table 1**). Past experience has shown that prior antimicrobial drug treatment reduces the chances of detecting leptospires in the blood by real-time PCR and culture (data not shown).

We collected demographic and epidemiologic data (age, sex, gender, occupation or hobbies, exposure to water and/or animals) of the twenty-two leptospirosis patients. Many patients were male (77.2%). The median age was 34 (13–78) years old. Patients originated from various locations, illustrating the fact that this disease is distributed across the whole country. Most cases (16/22) occurred during the hot and rainy season from December to April (temperate dry season from May to October). Among the leptospirosis cases with information on occupation (6/22), all had occupations associated with contact with surface water or animals (**Table 1**).

The clinical manifestations of leptospirosis range from a mild febrile illness to a severe and potentially fatal illness, characterized by jaundice, renal failure, thrombocytopenia, and hemorrhage (Weil's disease). Two patients did not survive. Clinical data generally included fever, myalgia, headache, and elevated bilirubin, creatinine, and transaminase. Patients did not exhibit severe forms of pulmonary leptospirosis.

### Serological characterization of clinical isolates

Serological identification of the clinical isolates was performed using the microscopic agglutination test (MAT) method with reference sera that were representative of the major *Leptospira* serogroups (Text S1). Serogrouping of the 22 isolates revealed agglutination titers for antisera raised against serogroups Mini, Hebdomadis, Pyrogenes, and Grippotyphosa, and negative reactions with antisera raised against the remaining serogroups. The vast majority of isolates (20/22) exhibited high agglutination reactions (400–12,800) with the antiserum raised against serogroup Mini, of which 6/20 also showed detectable agglutination reactions (200–3,200) with the Hebdomadis antiserum. Strains 2008/01774 and 2007/01872 showed a high level of reaction with rabbit antiserum raised against serovars Grippotyphosa and Pyrogenes, respectively (**Table 1**).

### Molecular characterization of clinical isolates

**Figure 3:**
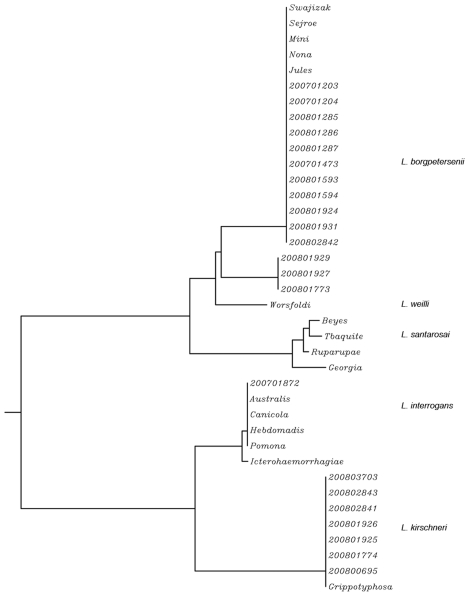
*ligB* gene sequence-based phylogeny of clinical isolates from Mayotte and representative serovars belonging to serogroups Mini, Hebdomadis, and Grippotyphosa. *L. interrogans* reference strains (serovars Australis, Icterohaemorragiae, Canicola, and Pomona) were also included in the dendrogram. The dendrogram was constructed by using the neighbour-joining method.

*Leptospira* isolates were characterized by partial 16S rRNA and *ligB* sequencing, the amplification of VNTR loci, and PFGE separation of *Not*I-digested genomic DNA (**Table 1**). The amplification and sequencing of the 16S rRNA gene (*rrs*) showed that clinical isolates belong to the pathogenic species *L. borgpetersenii* (14/22), *L. kirschneri* (7/22), and *L. interrogans* (1/22). Sequence analysis of *ligB*, which is a pathogen-specific gene [26], allowed differentiation below the species level. Thus, *L. borgpetersenii* isolates are further divided in two different clusters (A and B). The dendrogram showing all *ligB* genotypes is shown in **Figure 3**.

MLVA (Multi Locus VNTR Analysis) has been mainly developed for the pathogens *L. interrogans* and *L. kirschneri* [27]. The locus VNTR 7 is not found in *L. borgpetersenii*; thus, two other loci (Lb4 and Lb5) are usually amplified for typing *L. borgpetersenii* isolates [27]. The five patterns defined by MLVA are in agreement with the four clusters determined by *ligB* sequencing (**Table 1**). None of these genotypes has been previously identified in reference strains when compared to members of serogroups Mini, Hebdomadis, Pyrogenes, and Grippotyphosa [27].

**Figure 4:**
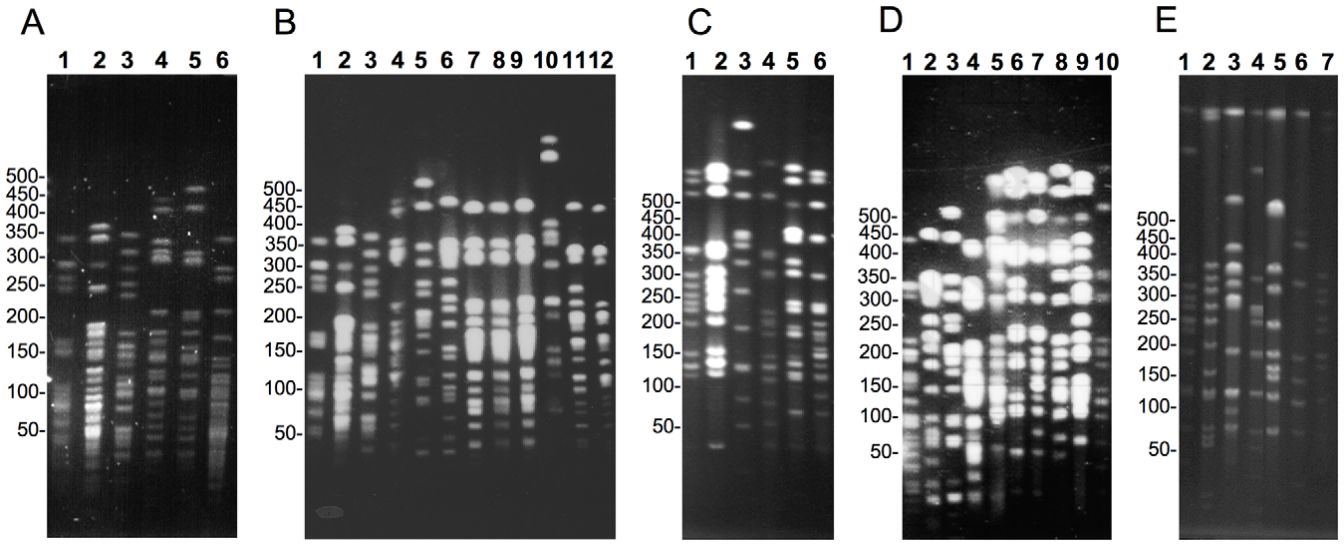
PFGE separation of *Not*I-digested genomic DNA from *Leptospira* isolates. **A**. Reference strains from serogroup Mini: serovars Beye (1), Georgia (2), Ruparupae (3), Mini (4), Swajizak (5), and Tbaquite (6). **B**. serovar Beye (1), serovar Georgia (2), serovar Ruparupae (3), serovar Mini (4), 2008/01927 (5), 2008/01929 (6), 2008/01931 (7), 2008/02842 (8), 2008/01924 (9), serovar Kambale (10), serovar Nona (11), and serovar Jules (12). **C**. serovar Grippotyphosa strain Valbuzzi (1), serovar Grippotyphosa strain Moskva V (2), serovar Grippotyphosa strain Andaman (3), 2008/01774 (4), 2008/03703 (5), 2008/00695 (6). **D**. serovar Jules (1), 2008/01773 (2), 2008/01927 (3), 2008/01926 (4), 2008/02843 (5), 2008/03703 (6), 2008/00695 (7), 2008/01925 (8), 2008/02841 (9), 2008/01774 (10). **E**. 2007/01872 (1), serovar Abramis (2), serovar Biggis (3), serovar Camlo (4), serovar Guaratuba (5), serovar Manilae (6), and serovar Pyrogenes (7). The molecular weight size marker consisted of bacteriophage lambda DNA concatemers of 50 kb.

PFGE is the long-standing gold standard method for genotyping *Leptospira* strains [29], [30], [31]. Tenover *et al.* [32] proposed PFGE criteria for interpreting the relatedness of epidemiologically related bacterial isolates. In addition, several studies have shown that PFGE profiles for *Leptospira* strains of a serovar belonging to the same species were indistinguishable or closely related [29], [30], [31]. In this study, isolates were considered different if more than three band differences were observed. Eleven of 21 isolates (52%) generated a common pattern (pattern I) with PFGE separation of *Not*I-digested genomic DNA. Thus strains isolated from different patients over a one-year period (from April 2007 to June 2008) gave indistinguishable PFGE patterns. The other 10 isolates were grouped into at least six other clusters. Strains 2008/0695, 2008/01926, 2008/03703, the “Grippotyphosa” (strain 2008/01774) and “Pyrogenes” (strain 2007/01872) isolates all displayed a unique PFGE pattern (**Figure 4**).

MLVA and PFGE patterns were compared against fingerprints from members of serogroups Mini, Hebdomadis, Pyrogenes, and Grippotyphosa, as well as against three african strains belonging to serogroups Grippotyphosa, Sejroe, and Hebdomadis [33], [34] (Text S1).

The PFGE and MLVA patterns obtained for *Leptospira* clinical isolates that reacted with rabbit antisera raised against serovar Mini were quite different from the patterns obtained for reference strains of the serogroups Hebdomadis (serovars Jules, Nona, Kabura, Kambale, Kremastos, Worsfoldi, and Hebdomadis) and Mini (serovars Mini, Beye, Georgia, Swajizak, Ruparupae, and Tbaquite) (**Figure 4**). An interesting observation is the similarity of the PFGE reference strain restriction patterns for serovars Jules and Nona (serogroup Hebdomadis), which are *L. borgpetersenii* isolates of African origin (Text S1) (**Figure 4**). These two serovars were also indistinguishable by MLVA (data not shown). Similarly, serovars Mini and Swajizak (*L. borgpetersenii* strains belonging to serogroup Mini) appeared to be closely related based on their PFGE restriction patterns (**Figure 4**). The “Grippotyphosa” strain displayed a genotype that was different to that displayed by others members of the serogroup Grippotyphosa (serovars Grippotyphosa, Canalzanae, Dadas, Valbuzzi, Vanderhoedeni, Ratnapura, Muelleri, and Liangguang) (**Figures 4** **&** **5**). Similarly the “Pyrogenes” strain was different from the MLVA (data not shown) and PFGE (**Figure 4**) patterns of members that belong to the *L. interrogans* serogroup Pyrogenes (serovars Pyrogenes, Abramis, Camlo, Guaratuba, Manilae, Robinsoni, Biggis, and Zanoni).

**Figure 5:**
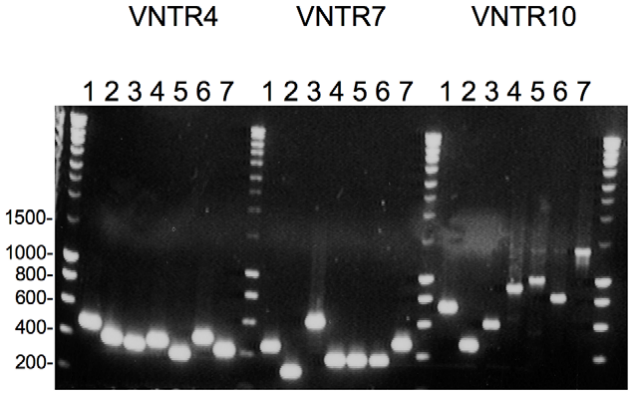
PCR analysis of the polymorphism of VNTR4, VNTR7, and VNTR10 loci of “Grippotyphosa” strains. Lanes 1: serovar Muelleri, 2: serovar Liangguang, 3: serovar Ratnapura, 4: serovar Vanderhoedeni, 5: serovar Grippotyphosa strain DF, 6: serovar Grippotyphosa strain Moskva V., 7: strain 2008/01774.

### Animal experiments

**Figure 6:**
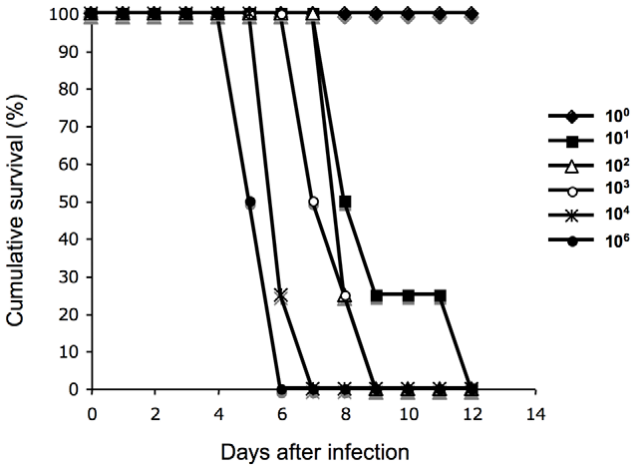
Virulence of clinical isolate 2007/01203 in the gerbil model of leptospirosis. Infection experiments with strain 2007/01203 were performed with four gerbils per group. Groups of gerbils were inoculated with 10^6^, 10^4^, 10^3^, 10^2^, 10^1^, and 10^0^ leptospires.

**Figure 7:**
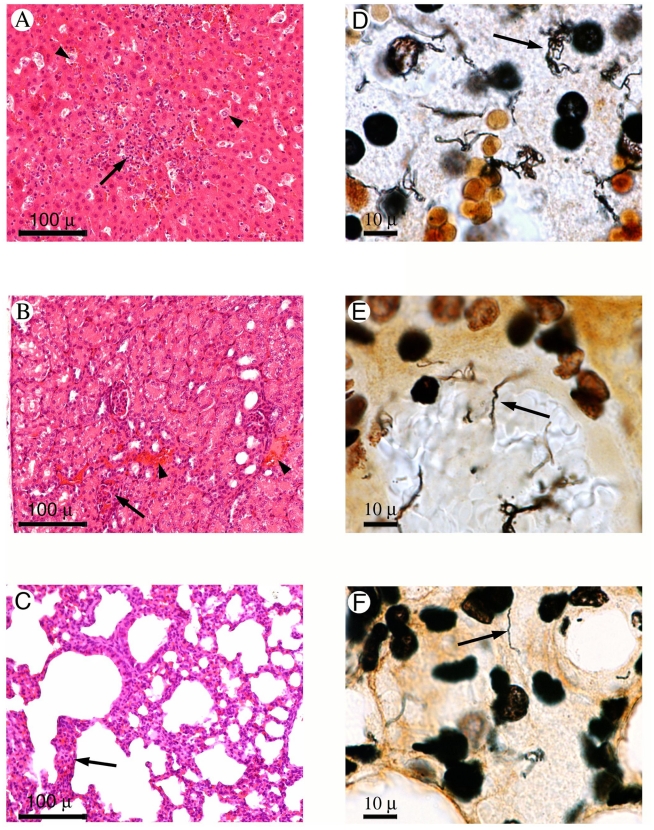
Histopathological sections of liver (A, D), kidney (B, E), and lung (C, F) samples from gerbils infected with clinical isolate 2007/01203. Left panel: Hematoxylin and eosin staining (×100). Right panel: Whartin Starry staining (×1000). Hepatocyte necrosis (A, arrowhead) and Kupffer cells surrounded by polymorphonuclear and mononuclear cells were observed within the medio lobular zones of the liver (A, arrows). Periportal infiltrates with lymphocytes were also observed. Several spirochetes were distributed around the hepatocytes (D, arrow). Kidneys exhibited hemorrhaging around the tubules (B, arrowhead), necrosis of tubular epithelia, and interstitial nephritis with infiltrates of lymphocytes, and monocytes, and occasional polymorphonuclear cells (B, arrow). Spirochetes were observed within renal tubules, venular endothelia (E, arrow), and glomeruli. Infiltrates with polymorphonuclear and mononuclear cells were seen in alveolar septa (C, arrow). Spirochetes were found in the capillary network of the alveolar septa (F, arrow).

A representative strain of the major genotype (2007/01203) was tested for virulence in the gerbil infection model of acute leptospirosis [35], [36]. Inoculation with as low as 10 bacteria of strain 2007/01203 induced death in 100% of the animals. Infected gerbils died within 5 to 10 days after the infection (**Figure 6**). Nasal bleeding was frequently observed in infected animals just before they died. Although gross hemorrhages or jaundice were not detected during necropsy, microscopic foci of hemorrhages were detected in the liver, kidneys, and lungs (**Figure 7**). Silver impregnation demonstrated a large numbers of spirochetes in the livers, kidneys, and lungs of infected gerbils (**Figure 7**).

## Discussion

The characterization of *Leptospira* isolates is essential if we are to understand the epidemiological properties of the disease. *Leptospira* serovars usually demonstrate specific host preferences. For example, rats serve as reservoirs for the serogroup Icterohaemorragiae, whereas house mice are the reservoir for the serogroup Ballum [1]. Local *Leptospira* isolates may also serve as antigens for the serodiagnosis of leptospirosis. However the isolation of *Leptospira* from blood specimens is usually rare because of the low sensitivity of the technique, the need for specific medium (EMJH) and a prolonged period of incubation (>1 month) [37]. Infection causes a leptospiremia within the first week of illness until the host mounts an effective acquired immune response, leaving a narrow window in which bacteria can be detected in blood. By screening blood from patients with febrile illness by real-time PCR, we were able to obtain a high rate of isolation of *Leptospira*. The real-time PCR technique has been shown to provide good sensitivity and a linear relationship between the bacteria copy number and cycle threshold (Ct) values. In this study, blood samples from the two deceased patients exhibited the lowest Ct values (corresponding to 1×10^5^ and 7.7×10^5^ leptospires per ml of blood, respectively) (**Table 1**). It was previously shown that a density of 10^4^ leptospires per ml of blood is a critical threshold for the vital prognosis of patients [38], [39].

In this study, we isolated 22 strains from the blood of human leptospirosis cases in Mayotte. The isolates were pathogenic to gerbils and were identified by serology and molecular typing. Based on serological data, serogroup Mini appears to be the dominant cause of leptospirosis in Mayotte. However, the first infections due to serogroup Mini were only reported in 2007, as culture isolation techniques were not attempted for logistic reasons in Mayotte before 2007. Prior to 2007, the standard MAT was performed and it did not include the serogroup Mini in its panel of antigens. Over the last ten years (1998–2008), both serological characterization of isolates (since 2007) and serological detection of antibodies in patient sera have shown that the most prevalent *Leptospira* serogroups in Mayotte have been Sejroe (21%), Grippotyphosa (14%), and Pyrogenes (10%). Serogroup Icterohaemorrhagiae accounted for only 5% of cases (12 of 230 patient sera) (data from the National Reference Center of Leptospira, France). Three isolates were identified as members of the serogroup Mini in 2007 and 16 isolates were identified as members of the serogroup Mini in 2008. In 2009, 84 cases have been diagnosed by PCR, from which 41 positive cultures were identified at the serological level. The most prevalent *Leptospira* serogroups were Mini (28/41), Pyrogenes (2/41), Pomona (8/41), and Grippotyphosa (1/41).

The serovar Mini was originally isolated from a patient in Italy in 1940. Other serovars were subsequently isolated from humans and animals (opposum, bandicoot, raccon) and classified into the serogroup Mini (Text S1) as a function of their antigenic determinants [18]. Historically, members of serogroup Mini belonged to the larger serogroup Hebdomadis [18]. This serogroup was then divided into three autonomous serogroups: the serogroups Hebdomadis, Mini, and Sejroe. Members of these serogroups may therefore exhibit serological affinities [18] as observed between serogroups Mini and Hebdomadis for some of our clinical isolates (**Table 1**).

Four molecular typing methods have been used to further characterize the 22 *Leptospira* clinical isolates. The four molecular typing methods revealed independent polymorphisms (**Table 1**). 16S ribosomal RNA gene sequence analysis can distinguish leptospiral species, but it is unable to distinguish between serovars. *ligB* sequencing and MLVA identified four and five genotypes, respectively, whereas PFGE allowed the recognition of subgroups within these genotypes. Thus, PFGE was the best method for discriminating between strains among the tested typing methods; moreover, it showed a strong correlation with the other techniques. However, the rapidity of PCR makes MLVA and *ligB* sequencing appropriate for the preliminary genotyping of clinical isolates [26], [27].

The pathogens *L. borgpetersenii* and *L. kirschneri* were the dominant species among clinical isolates in Mayotte (located between Tanzania and Madagascar). These data are in agreement with previous studies that show that *Leptospira* isolates from Africa belong to either *L. borgpetersenii* or *L. kirschneri* species [33], [34], [40], [41]. By contrast, in other areas, *L. interrogans* is considered to be the major species responsible for human infections. Interestingly, the serovars belonging to serogroup Mini were found in clinical isolates belonging to both *L. borgpetersenii* and *L. kirschneri*. The “serovar” is identified based on structural heterogeneity of the O-antigen, which is the carbohydrate component of the lipopolysaccharide (LPS). Horizontal gene transfer of the LPS locus between different *Leptospira* species may be responsible for the presence of serovars with serologically related LPS in various species. Thus, the LPS locus of the antigen-related serovars Hardjobovis and Hardjoprajitno belonging to species *L. borgpetersenii* and *L. interrogans*, respectively, are highly similar [42].

Genomic macrorestriction with *Not*I followed by PFGE is considered to be a powerful typing method for classifying *Leptospira* strains at the serovar level [29], [30], [31], [43]. For example, classification of the Dadas I strain as a new serovar of serogroup Grippotyphosa was strongly supported by its unique pulsed-field gel electrophoresis pattern [43]. The clinical isolates from Mayotte had PFGE patterns that were different to those for the reference strains belonging to serogroups Mini, Hebdomadis, Pyrogenes, and Grippotyphosa (Text S1). These data were confirmed by MLVA. Non tested reference serovars (not present in our collection of strains) include *L. weilii* serovar Hekou and *L. interrogans* serovar Perameles [44] from serogroup Mini, *L. noguchi* serovar Huanaco from serogroup Grippotyphosa, and *L. santarosai* serovars Borincana, Goiano, Maru, and Sanmartini and the *L. alexanderi* serovar Manzhuang from the serogroup Hebdomadis [18]. However none of these serovars belong to the identified *Leptospira* species (i.e. *L. borgpetersenii* and *L. kirschneri*) and may therefore be phylogenetically distant from our clinical isolates.

Our findings suggest that clinical isolates belonging to PFGE patterns I to X may represent ten new serovars. These isolates are highly virulent as observed in experimental animals (LD_50_<10 leptospires for strain 2007/01203) and are associated with severe clinical forms that could lead to death (2 of the 22 patients). These results highlight the potential existence of several undiscovered *Leptospira* serovars in the Indian Ocean. Further characterization of these isolates should include the use of the cross agglutination absorption tests (CAAT) to determine whether these isolates correspond to previously unknown serovars [45].

A previous survey in Madagascar found no antibodies to leptospirosis or the presence of pathogenic leptospires in possible animal reservoirs or in humans in contact with these animals [46]. Interestingly, patient 20 (a 52 year-old male) reported swimming in a river in Madagascar 10 days prior the onset of symptoms (and no other risk activities during this period). Since the incubation period of the disease is usually 5–14 days, the relationship between symptoms and the water exposure suggests that this traveler acquired the disease in Madagascar, a country with no prior reports of leptospirosis. The strain (2008/03703) isolated from this patient has been grouped with the *L. kirschneri* serogroup Mini isolates from Mayotte. However, it does exhibit a unique genotype by PFGE (**Table 1**).

Since the population in Mayotte is primarily of African origin, the epidemiology of leptospirosis in Mayotte may be the result of the introduction of pathogenic strains coming from the neighboring African countries as in the case of the chikungunya [47] and Rift Valley fever [48] outbreaks in Mayotte. Apart from the serogroups Hebdomadis and Grippotyphosa already reported in Africa, there are no references, to our knowledge, to strains belonging to the serogroup Mini in the African continent.

The high incidence of leptospirosis in Mayotte may be explained by the risk of exposure to infected animals. Contact with ruminants (sheep, cattle, or goats) is frequent. Dogs and rats (*Rattus rattus*; Norway rats or *Rattus norvegicus* have never been described in Mayotte) are usually encountered in the household area. Mayotte has also an abundance of endemic fauna such as the maki, a type of lemur, and the roussette (flying fox), a large bat. In 1991, cattle, herd, dogs and the common tenrec (*Tenrec ecaudatus*), a small spiny insectivorous mammal that resembles a hedgehog also found in Madagascar and other islands of the Indian Ocean, were serologically surveyed (88 serum samples) for leptospirosis using MAT [49]. In total, between 50 and 87% of the samples for each group of animals were seropositive for at least one *Leptospira* serogroup. The most prevalent *Leptospira* serogroups were Grippotyphosa, Sejroe, Icterohaemorrhagiae, Pyrogenes, and Canicola. Although MAT testing was unable to detect antibodies to *Leptospira* in the rat sera, a more recent study did detect leptospira (5 positive samples out of 27) in rat kidneys by PCR (unpublished data). These findings demonstrate that these animals are constantly exposed to *Leptospira* in their environment. The high prevalence of leptospiral infection in animals represents a potential threat to human health.

In summary, we identified 10 potentially new pathogenic *Leptospira* genotypes. Further ecological and surveillance studies are needed in Mayotte to identify the reservoir host(s) involved in transmission and to determine the public health impact and distribution of pathogenic leptospires in the region.

## Supporting Information

Text S1 List of *Leptospira* serogroup antisera used to characterize *Leptospira* clinical isolates.
(0.14 MB DOC)
