# Supplementary material for: Isolation and Characterization of New Leptospira Genotypes from Patients in Mayotte (Indian Ocean)
Source: PLoS Negl Trop Dis. 2010 Jun 22;4(6):e724. doi: 10.1371/journal.pntd.0000724 (PMC2889827; doi:10.1371/journal.pntd.0000724)
Supplement: Text S1 — List of Leptospira serogroup antisera used to characterize Leptospira clinical isolates. (0.14 MB DOC) [file pntd.0000724.s001.doc]

| **Suppl. Table 1: List of *Leptospira* serogroup antisera used to characterize *Leptospira* clinical isolates** | | | | | |
| --- | --- | --- | --- | --- | --- |
|  |  |  |  |  |  |
| Serogroup | Serovar | Strain |  |  |  |
| Australis | Australis | Ballico |  |  |  |
| Autumnalis | Autumnalis | Akiyami A |  |  |  |
| Bataviae | Bataviae | Van Tienen |  |  |  |
| Canicola | Canicola | Hond Utrecht IV |  |  |  |
| Ballum | Castellonis | Castellon 3 |  |  |  |
| Cynopteri | Cynopteri | 3522C |  |  |  |
| Grippotyphosa | Grippotyphosa | Moskva V. |  |  |  |
| Sejroe | Hardjo | Sponselee |  |  |  |
| Hebdomadis | Hebdomadis | Hebdomadis |  |  |  |
| Icterohaemorrhagiae | Copenhageni | M20 |  |  |  |
| Panama | Panama | CZ 214 K |  |  |  |
| Semaranga | Patoc | Patoc 1 |  |  |  |
| Pomona | Pomona | Pomona |  |  |  |
| Pyrogenes | Pyrogenes | Salinem |  |  |  |
| Sejroe | Sejroe | M 84 |  |  |  |
| Tarassovi | Tarassovi | Mitis Johnson |  |  |  |
| Icterohaemorrhagiae | Icterohaemorrhagiae | Verdun |  |  |  |
| Celledoni | Celledoni | Celledoni |  |  |  |
| Djamisan | Djasiman | Djasiman |  |  |  |
| Mini | Mini | Sari |  |  |  |
| Sarmin | Sarmin | Sarmin |  |  |  |
| Shermani | Shermani | 1342 K |  |  |  |
| Javanica | Javanica | Veldrat Batavia 46 |  |  |  |
| Louisiana | Louisiana | LSU 1945 |  |  |  |
|  |  |  |  |  |  |
|  |  |  |  |  |  |

| **Suppl. Table 2: Reference strains used in this study** | | |  |  |  |
| --- | --- | --- | --- | --- | --- |
|  |  |  |  |  |  |
| Species | Serogroup | Serovar | Strain | Country | Source |
| *L. interrogans* | Pyrogenes | Pyrogenes | Salinem | Indonesia | Human |
| *L. interrogans* | Pyrogenes | Abramis | Abraham | Malaysia | Human |
| *L. interrogans* | Pyrogenes | Biggis | Biggs | Malaysia | Human |
| *L. interrogans* | Pyrogenes | Camlo | LT 64-67 | Vietnam | Human |
| *L. interrogans* | Pyrogenes | Guaratuba | An 7705 | Brazil | Opossum |
| *L. interrogans* | Pyrogenes | Manilae | LT 398 | Philippines | Rat |
| *L. interrogans* | Pyrogenes | Robinsoni | Robinson | Australia | Human |
| *L. interrogans* | Pyrogenes | Zanoni | Zanoni | Australia | Human |
| *L. borgpetersenii* | Hebdomadis | Jules | Jules | Zaire | Human |
| *L. borgpetersenii* | Hebdomadis | Nona | Nona | Zaire | Human |
| *L. kirschneri* | Hebdomadis | Kabura | Kabura | Zaire | Human |
| *L. kirschneri* | Hebdomadis | Kambale | Kambale | Zaire | Human |
| *L. interrogans* | Hebdomadis | Kremastos | 2414 VAB | Peru/Panama | Human |
| *L. interrogans* | Hebdomadis | Hebdomadis | Hebdomadis | Japan | Human |
| *L. weilii* | Hebdomadis | Worsfoldi | Worsfold | Malaysia | Human |
| *L. borgpetersenii* | Mini | Mini | Sari | Italy | Human |
| *L. borgpetersenii* | Mini | Swajizak | Szwajizak | Australia | Human |
| *L. santarosai* | Mini | Beye | 1537 U | Panama | Spiny rat |
| *L. santarosai* | Mini | Georgia | LT 117 | USA | Racoon |
| *L. santarosai* | Mini | Tbaquite | TRVL 3214 | Trinidad | Human |
| *L. kirschneri* | Grippotyphosa | Grippotyphosa | Moskva V. | Russia | Human |
| *L. kirschneri* | Grippotyphosa | Grippotyphosa | Duyster | The Netherlands | Human |
| *L. kirschneri* | Grippotyphosa | Vanderhoedeni | Kipod 179 | Israel | Hedgehog |
| *L. kirschneri* | Grippotyphosa | Grippotyphosa | DF | USA | Human |
| *L. kirschneri* | Grippotyphosa | Ratnapura | Wumalasena | Sri Lanka | Human |
| *L. kirschneri* | Grippotyphosa | Dadas | Dadas I | Turkey | Bull |
| *L. interrogans* | Grippotyphosa | Muelleri | RM2 | Malaysia | Rat |
| *L. interrogans* | Grippotyphosa | Liangguang | 1880 | China | Rat |
| *L. interrogans* | Grippotyphosa | Valbuzzi | Valbuzzi | Australia | Human |
| *L. interrogans* | Grippotyphosa | Grippotyphosa | Andaman | Andaman Islands | Human |
| *L. santarosai* | Grippotyphosa | Canalzonae | CZ 188 | Panama | Spiny rat |
| "Zimbabwe" isolates: | |  |  |  |  |
| *L. borgpetersenii* | Hebdomadis | Marondera | SBF5 | Zimbabwe | Bovine |
| *L. borgpetersenii* | Sejroe | Hardjobovis | SBF31 | Zimbabwe | Bovine |
| *L. kirschneri* | Grippotyphosa | ND | SBF32 | Zimbabwe | Bovine |
|  |  |  |  |  |  |
|  |  |  |  |  |  |
| ND: not determined |  |  |  |  |  |
